# Data Sharing Mechanism of Sensors and Actuators of Industrial IoT Based on Blockchain-Assisted Identity-Based Cryptography

**DOI:** 10.3390/s21186084

**Published:** 2021-09-10

**Authors:** Yifan Meng, Jingzhao Li

**Affiliations:** College of Electrical and Information Engineering, Anhui University of Science & Technology, Huainan 232000, China; ivanmeng@outlook.com

**Keywords:** Industrial IoT, identity-based cryptography, device sharing, blockchain-assisted

## Abstract

The Industrial IoT is one of the key technologies to improve industrial production efficiency. The entire production process usually involves multiple production regions and numerous smart devices (sensors and actuators). The efficiency of the Industrial IoT is limited by this strong coupling relationship between the subsystem and the sensors and actuators. In this paper, to unleash the potential of Industrial IoT, a safe and reliable data sharing mechanism of sensors and actuators is proposed. We deployed distributed identity authentication and data proxy services in various regions. In the device authentication process, we used identity-based encryption algorithms, and we solved the trust problem between different regions by introducing a private blockchain. In addition, we designed the model of device capability (MDC) to describe the device, enabling it to be shared with a standard interface. Finally, we conducted many performance tests on the proposed mechanism. The test results verified the effectiveness and efficiency of the proposed mechanism.

## 1. Introduction

The core feature of Industry 4.0 is the information interconnection of the entire production process. Thus, the key to industry production entering the era of Industry 4.0 is to speed up the development of information physics systems. Enterprises deploy a large number of sensors and actuators in each production process, but these devices can only act on the subsystem to which they belong. The strong coupling between devices and subsystems limits the effectiveness of the Industrial Internet of Things (I-IoT).

In the engineering practice of industrial production companies represented by coal mines, we always find it difficult to call the data of the company’s existing sensors and actuators. Obviously, this restricts the efficient operation of the I-IoT.

Although terminal devices can communicate with each other from the perspective of network topology, establishing a secure communication channel with a controllable identity is one of the issues that need to be addressed.

Unprotected exposure of terminal equipment to the system will cause serious security vulnerabilities. This is because sensors and actuators can directly affect the physical world. For example, malicious people who can illegally control the actuators on the production line will cause serious accidents. It is necessary to establish a sharing mechanism for sensors and actuators. This mechanism needs to resolve these problems:

① Establish a secure and reliable communication link for visitors who access the sensors and actuators.
② Ensure that the identities of communication parties in the system are trusted.
③ Be compatible with heterogeneous devices.

To build a safe and reliable sharing mechanism of sensors and actuators in industrial IoT, we formulated the following design goals and principles:

① **Simple structure:** To ensure the efficiency of the mechanism, the structure should be relatively simple and new entities should not be introduced unnecessarily.
② **Good compatibility:** The mechanism should minimize the need for computing capacity of terminal devices and ensure its availability to capacity-constrained devices.
③ **Communication link security:** The mechanism should provide secure end-to-end communication and secure communication links for devices and visitors.
④ **Controllable access permissions:** The mechanism should refuse unauthorized user access and actively close the data link.
⑤ **Identity privacy protection:** The mechanism should ensure that the device identification information is not leaked and prevent malicious users from intercepting data packets to know what resources entities access.
⑥ **Credible identity:** The device should be trusted to ensure a reliable data source or control object. Visitors should be trusted to ensure that resources are not stolen by malicious third parties.

Low coupling between regions: The coupling between regions should be minimized to enhance the robustness of the entire system.

Guided by the above principles and objectives, we proposed a sharing mechanism of sensors and actuators (SMSA) based on a blockchain-assisted IBC (Identity-Based Cryptosystem). In this mechanism, terminal devices can safely and reliably share their capabilities. Through the model of device capability, visitors can call terminal devices in a standard way. Our contribution can be summarized as follows:

We proposed an efficient and secure sharing mechanism of sensors and actuators (SMSA) that can realize the ability sharing of various sensors and actuators in industrial IoT and give full play to the ability of various sensors and actuators in the network.

We constructed an identity authentication service based on blockchain-assisted IBC in SMSA to avoid maintaining many certificates in the system and improve the operation efficiency of the system.

The characteristics of sensors and actuators in mines were analyzed, and the capability description model of devices was designed to form Industrial IoT-distributed soft buses.

We conducted a safety analysis of the SMSA, then set up an experimental environment for performance evaluation. The test results showed that the performance of the SMSA computing overhead and data exchange delay met the requirements.

The rest of this paper is organized as follows. In the second section, we introduce the technical research work related to the sharing mechanism proposed in this paper. In the third section, we describe the problems to be solved and the technical background of the sharing mechanism. In Section 4, an overview of the mechanism is given, while the design details are given in Section 5. In Section 6, we describe our experiments to verify the effectiveness of the SMSA. Finally, the work is summarized in the seventh section.

## 2. Related Work

Sensor or actuator deployment performs fixed tasks. However, this deployment mode causes the sensors or actuators to be a specific sub-function of the Industrial IoT (I-IoT). When other tasks are needed to collect the same data, it is difficult to invoke existing sensor data, and this is often done by redeployment.

In the framework of cyber-physical convergence, industrial equipment will be generating large amounts of data for real-time control. Data in the Industrial IoT are becoming more and more important. Havlik et al. proposed simplifying service access data by building a standardized sensor data sharing platform [1]. They formatted the sensor data stream into a time series, which is convenient for visitors to call. Lim et al. pointed out that strong coupling between sensors and applications limits the availability of sensor data. To enhance the availability of sensor data, the team proposed building a flexible open platform. This platform can describe sensor capabilities and publish or subscribe to sensor data through standardized middleware [2]. To solve the problem of industrial wireless network communication data access in a multi-hop environment, Raptis et al. proposed a distributed data access method [3]. However, this model does not consider the issue of data access security. Sharing the data of various sensors in the network will challenge the security of each level of services. Malicious third parties can use tools similar to the Shodan [4] search engine to scan and obtain device control ports.

In the field of Industrial IoT security, researchers have done a lot of work. Borhani et al. summarized the security problems faced by the Industrial Internet of Things and possible solutions [5]. Gurtov and others analyzed the challenges faced by the Industrial Internet in the process of communication and data processing and proposed a secure communication architecture [6]. IoT systems already contain many kinds of authentication measures to protect communication safety. From the difference in encrypting keys, there are two categories of encryption algorithms: the symmetric encryption algorithm and the asymmetric cryptographic algorithm.

### 2.1. Encryption Algorithm

The symmetric encryption algorithm is a commonly used encryption method. The same key is used for encryption and decryption in the symmetric encryption algorithm [7]. The key is the command to control encrypt and decrypt processes. The algorithm is a group of rules that specify how to encrypt and decrypt. The safety of encryption depends on not only the algorithm but also the security management of the key is a decisive factor. Because of the simple structure of the symmetric encryption algorithm, it can encrypt or decrypt data at a high speed. This feature makes it suitable for scenarios where large amounts of data are transmitted. However, symmetric encryption algorithms are prone to security vulnerabilities during the key exchange process.

Message authentication code (MAC) [8] is a common technology in the symmetric encryption algorithm used to verify the identity of IoT devices. Wu et al. proposed ANA-MAC based on artificial-noise-aided [9]. Bellare et al. proposed nested construction (NMAC) and hash-based MAC (HMAC) [10]. HMAC hardware is integrated as a security measure in some embedded microprocessors. The problem of the symmetric key is a way to exchange keys safely [11].

Unlike symmetric encryption, asymmetric encryption does not require the exchange of all keys. The asymmetric cryptography algorithm encrypts the public key and private key through pairs, and the public key is visible to anyone [12]. Information encrypted by a private key can only be decrypted by the corresponding public key. Similarly, information encrypted by a private key can only be decrypted by the corresponding public key. In this case, encrypted communication is realized without exchanging all the keys, and the receiver can confirm that the message was sent by the private key holder. The encryption and decryption speed of asymmetric encryption algorithms is much slower than symmetric encryption algorithms [13]. This is because the structure of the asymmetric encryption algorithm is very complicated, which makes asymmetric encryption algorithms unsuitable for the transmission of large data volumes. Our basic idea is to build a secure key exchange channel between devices through an asymmetric encryption algorithm, then use a more efficient symmetric encryption algorithm to complete normal data encryption communication.

### 2.2. Certificate-Based Authentication

In the I-IoT, there are a large number of devices, and how to match the relationship between the key pair and the owner is one of the issues we needed to consider. The function of the certificate is to prove the mapping relationship between the key pair and the owner so that malicious third parties cannot fake the identities of other entities [14]. KumarVerma et al. proposed a certificate-based proxy signature scheme in the Industrial IoT scenario [15]. This solution provides data integrity authentication for the I-IoT at the lowest computational cost. Park designed a certificate-based security protocol for wireless mobile communication equipment [16]. In this protocol, an off-line authentication mechanism based on a dynamic certificate is introduced to realize the end-to-end Internet identity authentication key exchange protocol.

Certificate-based authentication requires a third party trusted by all as the *certification authority* (CA). The CA is the core of the *Public Key Infrastructure* (PKI), and it is the organ responsible for issuing certificates, certification certificates, and managing issued certificates.

### 2.3. Identity-Based Authentication

The mapping relationship between the user and its public key is verified by a certificate. In this case, a lot of certificates are managed by the certification authority. In identity-based mechanisms, however, the identity of the users is their public key [17]. The CA is not necessary for an identity-based system.

Wang applied identity-based authentication to portable mobile cellular networks to provide secure and anonymous conference calls [18]. Based on an identity-based hierarchical model for cloud computing (IBHMCC) and corresponding encryption and signature schemes, Li designed a new cloud computing and service authentication protocol, which reduced the burden on the user side compared with SAP (SSL Authentication Protocol) [19]. However, the above work failed to optimize the design for the situation of weak terminals in I-IoT. The computing capabilities of various terminals in the I-IoT are different, and the mechanisms need to have good compatibility.

### 2.4. Innovation of the Paper

In this paper, we proposed a sharing mechanism of sensors and actuators (SMSA) based on blockchain and IBC. The IBC technology is used to authentication the identity of entities in the I-IoT. Our innovation can be summarized as follow:

(1) To improve the compatibility of the mechanism to weak terminals, we reduced the encryption and calculation links that need to be performed on the terminal side in the authentication mechanism.
(2) We designed sub-regional authentication management based on edge computing technology to provide low latency authentication services for users in each region. The authentication information of each region is synchronized by blockchain technology to ensure the independence of each region. In this structure, when a certain region is attacked, the security of other regions can be guaranteed.
(3) We designed the model of device capability (MDC) to unify the terminal device data calling interface.

## 3. Overview

In this section, we describe the application background of the SMSA in detail, including the status and problems of the I-IoT.

### 3.1. Industrial IoT Overview

The SMSA is designed with the characteristics of the I-IoT, so we first introduce the system structure and terminal equipment of the I-IoT.

#### 3.1.1. Industrial IoT Structure

The I-IoT is the nerve center connecting all aspects of the production site. Through the I-IoT, all kinds of equipment in the production site can work together [20]. Edge computing nodes can provide lower latency computing services because they are closer to sensors and actuators than cloud servers [21].

The I-IoT is a heterogeneous integration network, terminal devices access the network through various communication gateways [22]. The industrial network takes high-speed industrial ethernet composed of optical fiber as its backbone and follows the *Open System Interconnection* (OSI) network standard protocol [23,24]. There are also field buses such as RS-485 in industrial production. The I-IoT inherits web services from the architecture that represent transmission status [25]. Communication between devices uses the *Constrained Application Protocol* (CoAP) [26], *Message Queuing Telemetry Transport* (MQTT) [27], and other protocols designed for the IoT environment.

#### 3.1.2. Overview of Inductive Equipment for the Industrial IoT

In industrial production, a large number of sensors are needed to obtain the parameters of the environment and electromechanical equipment. As Table 1 shows, in the industrial production is represented by mines, and these sensors belong to different professional departments. The sensing devices include the time-driven sensor and the event-triggered sensor.

These sensors output signals through different interfaces, and some devices directly output analog signals without basic digital interfaces. For devices that do not have network communication capabilities, it is necessary to design heterogeneous fusion gateways to enable them to have secure communication capabilities.

Most I-IoT actuators are driven by *Programmable Logic Controllers* (PLCs). In addition to autonomous control based on sensor data, PLCs can also receive remote control commands via ethernet to drive the execution device.

### 3.2. Security Threat

There are a lot of security threats in the process of sharing sensors and actuators. These security threats are what we need to guard against in the process of designing the sharing mechanism.

#### 3.2.1. Safety Capability of Sensors and Actuators

Sensors and actuators of the industrial IoT are not all driven by powerful SoCs (systems on chip). Manufacturers use low-power microprocessors to save production costs and reduce power consumption. The ESP32 series of SoC launched by Espressif has WiFi and Bluetooth 5.0. It is often used as the main control chip for wireless sensors and actuators. ESP32 has built-in hardware encryption accelerators (such as AES-128/192/256, HMAC, Digital Signature, etc.). It has basic security protection capabilities. However, due to the limitations of hardware resources, it is difficult to implement more flexible security policies. Therefore, in the sharing mechanism, sensors and actuators only complete basic security protection operations, and more complex security strategies are completed by agent devices.

#### 3.2.2. Security Threat of the Industrial IoT

**Figure 1:**
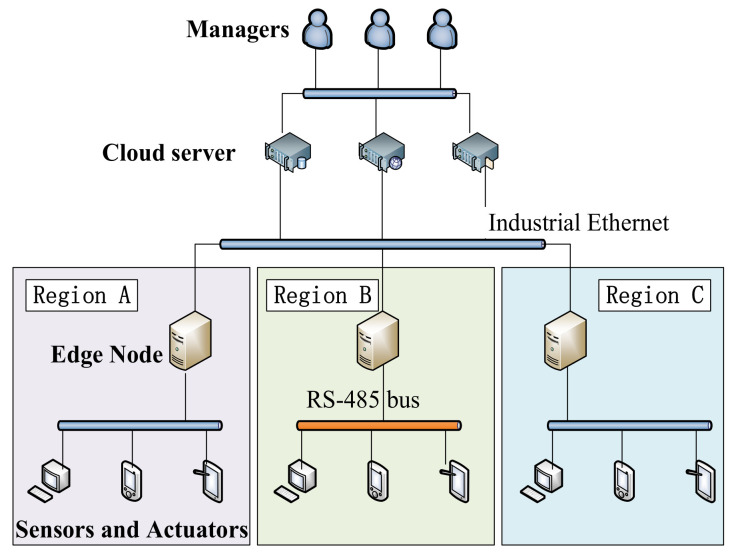
Typical architecture of the Industrial IoT.

In the architecture of the I-IoT shown in Figure 1, if the sensors and actuators are directly shared within the network, this will cause serious security threats.

(1) Privacy threat: Sensors and actuators are weak terminals. They cannot cope with sophisticated attack methods [28]. This means that if the interface of the terminal is directly exposed to the network, it will break the last layer of defense measures for the device. Attackers can get the privacy data by sniffer [29].
(2) Impersonate identity: A malicious third party will pretend to be an authorized user to gain control of the terminal device [30]. These devices are difficult to find within a short time of being hijacked because of their unattended features.
(3) Information congestion: To paralyze the industrial communication network, a malicious third party will use DDOS to attack key equipment in the network, causing information congestion [31]. This forms a distributed attack of denial service by infecting a large number of hosts with bot viruses [32].
(4) CAs compromised: In traditional identity authentication methods, a trusted third party will be introduced (such as a CA). However, a CA is vulnerable to attacks, and there is no way to ensure that a CA will not be tempted by huge profits to cause corruption.

## 4. Overview of the SMSA

In this section, we will introduce the solution of the SMSA and describe the main structure.

***A***. ***Structure of the SMSA***

**Figure 2:**
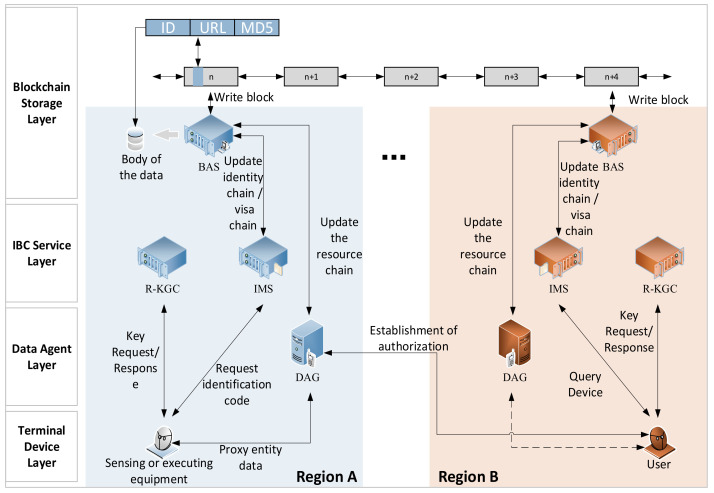
Structural diagram of the SMSA.

We designed the SMSA based on edge computing architecture to reduce latency in each region. As shown in Figure 2, the SMSA can be divided into four layers. The blockchain agent server (BAS), region key generate center (R-KGC), and identity management server (IMS) are all services running on edge computing facilities in each region. The data agent gateway (DAG) provides data agent services for various sensors and actuators in the region. The DAG and IMS of each region synchronize information through the BAS to help entity cross-region authentication.

The main private key of each R-KGC is independent. In this way, even if some R-KGCs’ main private keys are leaked, others’ main private keys are safe. In the SMSA, we chose to use identity-based signature (IBS) and the ephemeral elliptic curve Diffie–Hellman key exchange algorithm to construct the authentication and key agreement process.

***B***. ***Blockchain Storage Layer***

The blockchain storage layer is a distributed ledger composed of blockchain agent servers (BASs) in each region. The BAS needs to maintain three chains, namely, the identity management chain (I-Chain), the visa management chain (V-Chain), and the devices management chain (D-Chain).

**Figure 3:**
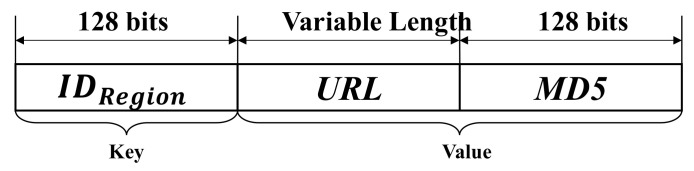
Format of the data written to the blockchain.

The BAS encrypts the data that need to be shared and stores it in the storage area of the BAS. Then it generates an index to point to the locations of these data, which will be written into the blockchain. In this way, the safe sharing of data between each region is realized, and the problem of slow blockchain IO speed is avoided. As shown in Figure 3, transaction data is written into blockchain with the format of key-value. The key is the identification of the region. Devices in other regions can quickly query data from the BAS by index $ID_{Region}$. The value includes two parts; one is the URL (Uniform Resource Locator) that points to where the data body is stored and the other is the MD5 value used to validate the data body.

By using the method of writing only index information in the blockchain without writing the data body, the amount of data that needs to be written to the blockchain can be effectively reduced. This data sharing method can shorten the time required for the BAS to write data.

***C***. ***IBC Service Layer***
  The IBC service layer includes the IMS and R-KGC of each region.
  (1) Identity management server (IMS): IMS is the facility that is used to manage the entity’s identity of each region. The entity sends the identity information form and the expected validity time to the IMS. The IMS generates a unique identification code (ID) for it. The ID is the identification of an entity in the system, and it is also the public key of the entity. The IMS stores and manages the identity information of entities and provides services for proxy verification of entity identities. After the IMS verifies the identity of the entity through the public key, it digitally signs the information sent outside the region to provide a credit guarantee. This process is called visa application. In this way, cross-region authentication is realized.
  (2) Region key generate center (R-KGC): The R-KGC is a virtual server running on edge computing facilities. The entity sends the ID obtained from the IMS to the R-KGC to request the generation of a private key. The R-KGC, IMS, and BAS of each region form a high-level region, and they share the same system parameters.
***D***. ***Data Agent Layer***

The data agent layer is composed of each DAG. The DAG is the core of data interaction in the SMSA mechanism, mainly used to help sensors and actuators send and receive data safely. The DAG distributes sensor data to all authorized demanders. At the same time, the instructions of the authorized visitors are passed to the executor. In this way, weak terminals such as sensors and actuators are not directly exposed to the network.

***E***. ***Terminal Device Layer***

The terminal device is composed of various underlying devices in the I-IoT, including sensors, actuators, and various smart devices.

## 5. Mechanism Details

In this section, we will introduce the proposed SMSA mechanism in detail.

### 5.1. Entity Identity Authorization 

The IBC is composed of four random algorithms, namely, Setup, Extract, Encrypt, and Decrypt. Before the system runs, running the setup algorithm first is necessary. After the setup algorithm runs, the system will be initialized, and the public parameters and main key will be returned. The public parameters will be made public, and the main private key is kept by the R-KGC.

Safety parameter k should be entered into the setup algorithm first. The cyclic addition group $G_{1}$ and cyclic multiplication group $G_{2}$ with order q are determined, and there is a bilinear map $e:G_{1}\times G_{1}\to G_{2}$. g is randomly generated as the generator of $G_{1}$. The R-KGC randomly selects a $s\in Z_{q}^{*}$ ($Z_{q}^{*}=\left\{0,1,2,\cdots ,q-1\right\}$) as the main private key, and the main public key is stated as $P_{pub}=sg$.

Two strong collision-resistant hash functions are defined: $H_{1}:{\left\{0,1\right\}}^{n}\to G_{1}$,$\;H_{2}:G_{1}\times {\left\{0,1\right\}}^{*}\to Z_{q}^{*}$. The function $H_{1}$ is used for mapping the ID to the cyclic addition group $G_{1}$. ${\left\{0,1\right\}}^{n}$ represents a set of binary sequence combinations of length n. The function $H_{2}$ is used to map a message of any length to an integer. ${\left\{0,1\right\}}^{*}$ is a set of sequence combinations of an arbitrary length. The public parameters of the system are: $params=\left\{k,q,G_{1},G_{2},g,e,H_{1},H_{2},P_{pub}\right\}$.

#### 5.1.1. Identity Management

**Figure 4:**
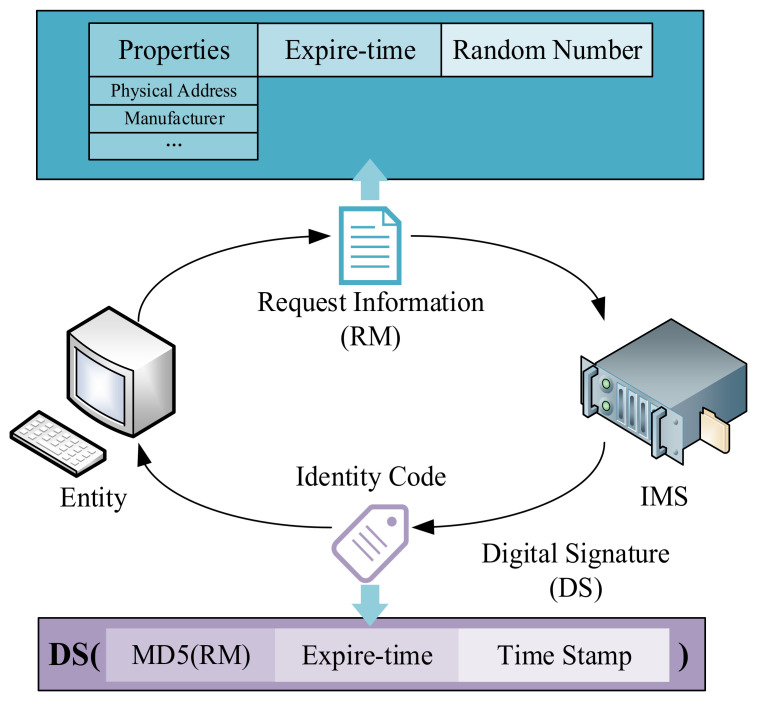
The process of entity requesting ID.

In the IBC system, the public key of an entity is its identification. This avoids the CA managing a large number of certificates. In the SMSA, to protect device identity information privacy, entities do not directly use their identity information as a public key but use the ID generated by the IMS as a public key. The process of entity requesting ID is shown in Figure 4.

The entity sends the ID request to the IMS. The request information includes the entity’s attribute, expire-time, and a random number. The attribute is the identity characteristics of the entity, such as the MAC address, manufacturer, serial number, etc. Expire-time is the valid period of the ID generated by the IMS. The random number is generated randomly by entities to mark request information.

After receiving the request, the IMS first stores the information in the database and updates the index stored in the identity management chain through the BAS. The IMS uses private keys to digitally sign MD5, expire-time, and current timestamps for request information. Finally, the signature result is returned to the entity as the ID.

The random number in the request is very important; even though a malicious third party can obtain various attributes of entities by illegal means, it is difficult to obtain the randomly generated verification code of entities. Therefore, when sending requests to the IMS and R-KGC for authentication, the entity needs to attach the random number of ID acquisitions as the security check code.

#### 5.1.2. Authentication Process

(1) Request Private Key

**Figure 5:**
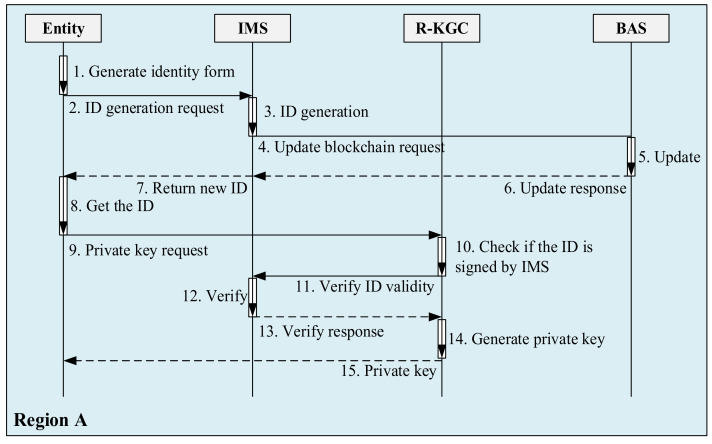
The process of entity requesting identity code and private key.

An entity needs to send a request to the R-KGC after obtaining an ID to get a private key. The process of entity requesting private keys is shown in Figure 5.

After receiving the request of entities, The R-KGC should first verify whether the ID was issued by the IMS through the IMS public key and check whether it is within the validity period. If the check is ok, the R-KGC will generate a private key for the entity. The entity’s private key generated by the R-KGC is $pk=sH_{1}\left(ID\right)$, the public key of the entity is $pk=H_{1}\left(ID\right)$.

(2) Local–Regional Authentication

Local–regional authentication is initiated by the party $e_{i}$ who claims its identity and verified by another part $e_{j}$. The authentication information of $e_{i}$ is named $Token_{ij}$. The format of $Token_{ij}$ is as follows:

(1) $$Token_{ij}=DS_{pk_{i}}\left(ID_{i}\left|\left|R_{i}\right|\right|T_{i}||Text\right)\left|\left|ID_{i}\left|\left|R_{i}\right|\right|T_{i}\right|\right|Text$$

where $DS_{pk_{i}}\left(X\right)$ is $e_{i}$ using its private key $pk_{i}$ to sign message X. The digital signature algorithm is shown as Algorithm 1. $ID_{i}$ is the identity of entity $e_{i}$, and it is also the public key of the entity. $R_{i}$ is a random number. $T_{i}$ is the timestamp when the token was generated. Text is the message that entity $e_{i}$ needs to pass to entity $e_{j}$ through $Token_{ij}$, and this field is not necessary.
**Algorithm 1.** IBC Digital Signature Algorithm**Input:** System parameters of the region *i*-th entity’s key pair $\left(pk_{i},sk_{i}\right)$, *j*-th entity’s public key $sk_{j}$, message *m*.$1:\;\mathrm{Random}\;\mathrm{selection}\;r_{1},r_{2}\in Z_{q}^{*}$;$2:\;\mathrm{Compute}\;R_{1}=r_{1}g$$,\;R_{2}=r_{2}sk_{i}$;$3:\;\mathrm{Compute}\;W_{1}=e\left(pk_{i},r_{2}sk_{j}\right)$;$4:\;\mathrm{Compute}\;S=r_{1}sk_{i}+pk_{i}H_{2}\left(W_{1},m\right)$;**Output:**$\;\mathrm{Signature}\;\sigma =\left(R_{1},R_{2},S\right)$.

After entity $e_{i}$ sends the $Token_{ij}$ to verifier $e_{j}$, $e_{j}$ uses the $ID_{i}$ in the $Token_{ij}$ as the public key to verify the digital signature and compare the information in the digital signature. The IBC digital signature verification algorithm is shown in Algorithm 2.
**Algorithm 2.** IBC Digital Signature Verification Algorithm **Input:** System parameters of the region *j*-th entity’s key pair $\left(pk_{j},sk_{j}\right)$, *i*-th entity’s public key $sk_{i}$$,\;Token_{ij}$.$1:\;\mathrm{Compute}\;W_{2}=e\left(sk_{i}pk_{j},r_{2}P_{pub}\right)$;$2:\;\mathrm{Compute}\;h=H_{2}\left(W_{2},m\right)$$3:\;\mathrm{if}\;e\left(S,g\right)=e\left(R_{1}+hP_{pub},sk_{i}\right)$:4: Verify correct;5: else:6: Verify error;

Because both authentication parties are entities in the same region, they have the same set of system parameters. In this case, the authentication operation can be completed without going through the IBC service layer.

(3) Cross-Regional Authentication

**Figure 6:**
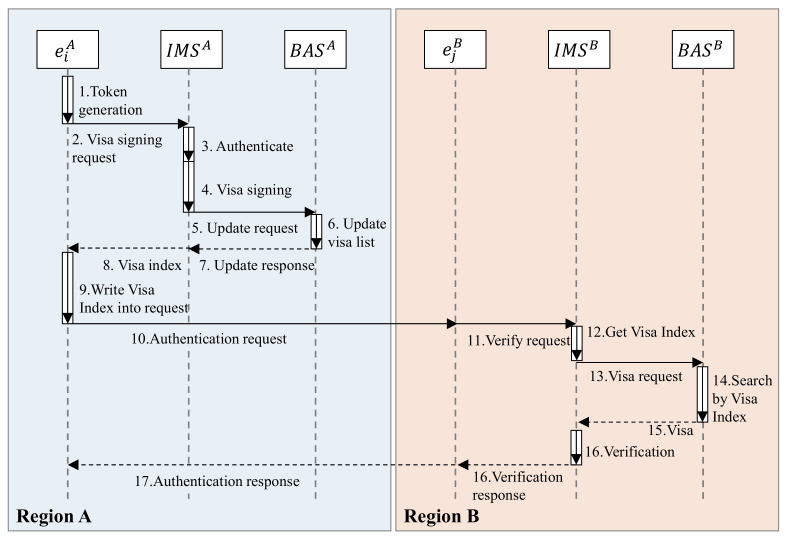
Cross-regional authentication process.

If the two parties are in different regions, they cannot share the same set of system parameters. Therefore, the signature and verification work cannot be completed directly, and the IBC service layer is required to participate in the completion. The cross-regional authentication is shown in Figure 6.

The entity $e_{i}^{A}$ in region A sends a token to $IMS^{A}$, and writes visa information into the Text field. $IMS^{A}$ will verify the token after receiving it. The signature algorithm and verify algorithm are the same as algorithms of local–regional authentication. When the verification is correct, $IMS^{A}$ issues a visa for the entity $e_{i}^{A}$. After that, $IMS^{A}$ requests $BAS^{A}$ to synchronize the visa to V-Chain. The format of the visa is:

(2) $$visa_{i}^{A}=E_{sk_{IMS^{B}}}\left(DS_{pk_{IMS^{A}}}\left(\mathrm{MD}5\left(ID_{i}\left|\left|R_{i}\right|\right|T_{i}||key\right)\right)||\left(ID_{i}\left|\left|R_{i}\right|\right|T_{i}||key\right)\right)$$

where $E_{sk_{DMS^{B}}}\left(X\right)$ means that $IMS^{B}$ uses its public key $sk_{DMS^{B}}$ to encrypt message X. $DS_{pk_{DMS^{A}}}\left(X\right)$ means that $IMS^{A}$ signs message X. $\mathrm{MD}5\left(X\right)$ generates the MD5 summary of message X. Key is the communication key after the entity $e_{i}^{A}$ is successfully authenticated. After the authentication is successful, the communication parties use symmetric encryption algorithms to ensure communication security and avoid the low throughput rate of asymmetric encryption. Finally, $IMS^{A}$ returns the index of the visa in V-Chain to entity $e_{i}^{A}$.

### 5.2. Entity Data Agent Process

To reduce the computing pressure on the terminal equipment, we used DAG to proxy the data transmission and reception of the terminal. In this section we will introduce the process of entity data agency.

#### 5.2.1. Device Capability Description Model

It is not only the security issue that hinders the sharing of sensors and actuators in the I-IoT, but the inconsistent data access interface is also one of the issues. Sensors and actuators in the Industrial IoT come from different manufacturers and belong to different departments. To reduce the complexity of data sharing caused by equipment diversity, the model of device capability (MDC) is established. The MDC summarizes and classifies sensors and actuators, and then abstracts a set of standard data structures and interfaces. The Industrial IoT can be regarded as a macro system composed of multiple distributed soft buses. These buses are divided according to the department to which the equipment belongs, and the visitor’s calling of data is simplified to read and write to the equipment on the soft bus.

**Figure 7:**
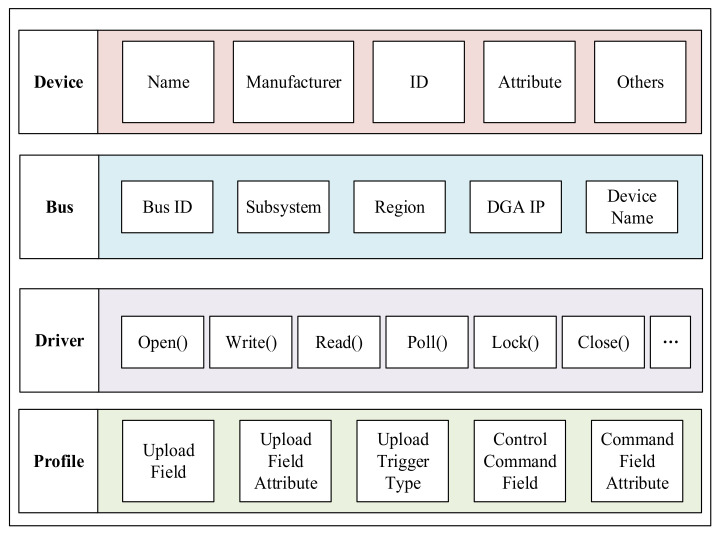
Structural diagram of the device capability description model.

As shown in Figure 7, the MDC describes the capabilities of the device from four aspects. It is similar to the device model of the Linux operating system.

The device part describes the basic information of the device, including fields such as the name, category, and attributes of the device. The bus part indicates on which soft bus the device is mounted. The driver part is a unified device abstract interface function. The device can implement a subset of the interface specified by the driver according to its capabilities. In this way, users can similarly access different devices. The profile part describes the function of the device, for example, what data the device can provide and the format of these data, what control commands the device supports and the format of the control commands. 

#### 5.2.2. Push Resource to DAG

If new equipment wants to share its resources in the mine IoT, it must first push its own MDC model to the DAG in its region. After the DAG receives the MDC model of the device, it first verifies the device according to the driver information described by the model. After the verification is passed, the device is mounted on the corresponding soft bus according to the bus information in the model. The mounting process is to insert the device model into the D-Chain according to the tree structure of “Bus→Region→DAG→Device.” Through this tree structure, visitors can quickly retrieve the device and obtain access information.

#### 5.2.3. Request DAG Authorization to Access a Resource

When a visitor in the I-IoT needs to call a device, it must first send a device retrieval request to the DAG in its region. After receiving the request, the DAG accesses the D-Chain through the BAS and retrieves the target device from the bus information according to the tree structure of “Bus→Region→DAG→Device.” The DAG returns the MDC model of the target device to the visitor. The visitor then applies for the access right of the target device corresponding to the DAG according to the information in the MDC model. If the visitor cannot complete the above operations independently, it can be handed over to the superior DAG agent.

After the visitor is authorized, it can receive real-time sensing device data pushed by the DAG and can also send control commands to a device through the DAG.

## 6. Performance Evaluation

Due to the different design ideas and applicable scenarios, the SMSA mechanism designed in this article is difficult to directly compare with other existing mechanisms. Therefore, in this section, we mainly focus on testing the security, computing performance, communication overhead, etc., of the proposed mechanism, and analyze the data to evaluate the feasibility.

In the system, the devices that need to perform secure operations include the DAG, IMS, R-KGC, and various entities. We implemented the IBC certification in the sharing mechanism based on the SM9 (national standard of China) issued by the State Cryptography Administration Office of Security Commercial Code Administration. The SM9 has been proven to be sufficiently safe for commercial use.

### 6.1. Experimental Setup

**Figure 8:**
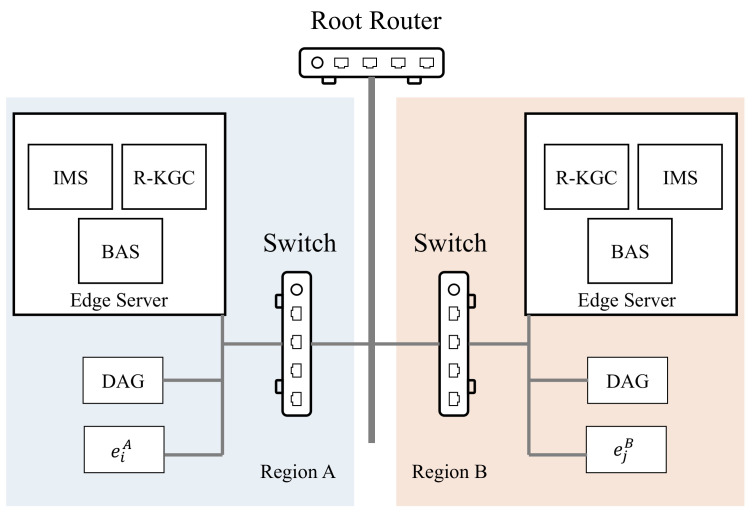
An illustration of the network topology in experiments.

In the experimental setup, we imitated the actual network environment of the mine and simulated an industrial IoT with two regions. The topology of the experimental network is shown in Figure 8. In each region, we deployed one desktop computer as an edge computing facility. The IMS, R-KGC and BAS, were all hosted on desktop computers as virtual machines using VMware Workstation 15 Pro. The hardware parameters of the desktop computer were AMD Ryzen7 5800X 3.8 GHz and 32 GB DDR4-3200 MHz RAM. The DAG was executed on a laptop with Intel Core i7-8550U and 8 G DDR4-2400 MHz RAM. The IoT devices were executed on a low-power industrial computer with Intel N3700 and 2 GB DDR3-1600 MHz RAM. The specific parameters of the IMS, R-KGC, BAS, DAG, and IoT devices are shown in Table 2. All virtual machines were enabled with Intel VT-x/AMD-V and connected to the LAN in bridge mode.

The communication between each device was based on CoAP (Constrained Application Protocol) [33]. CoAP was in binary format, HTTP was in text format, and CoAP was more compact than HTTP.

### 6.2. Performance Evaluation

We mainly evaluated the performance of the SMSA from two aspects, encryption operation and data interaction, and verified its feasibility. At the same time, the performance was compared with other existing mechanisms.

#### 6.2.1. The Performance of the Encryption Operation

At first, we conducted a theoretical analysis of the encryption operation of each process in the SMSA and evaluated the computational cost. The SMSA included the IMS, R-KGC, BAS, DAG, and IoT devices in each region. They cooperated to realize the safe sharing of sensors and actuators. Various types of devices performed different types of encryption operations in different workflows. As shown in Table 3, we sorted out the various encryption algorithms. Among them, *xor* means exclusive OR operation, pair means bilinear operation, *hash* means hash operation, and *exp* means exponential power operation.

It should be noted that the calculation process shown in Table 3 was only the signature and encryption scheme used in this experiment, and the SMSA mechanism did not force the use of a certain IBC scheme. Therefore, the performance evaluation results only carried out a feasibility analysis of the scheme used in the experiment. The encryption operation test program was based on the MIRACL (Multiprecision Integer and Rational Arithmetic C/C++ Library) function library [34] written in C++, and the *gcc* version of the Ubuntu operating system was 9.3.0. Table 4 shows the time taken by various facilities to execute each encryption algorithm. The length of the test message was 10,000 bytes, and the test was performed 1000 times to get the average value. From the test results, for IoT devices with weaker performance, the encryption algorithm took a long time to run. Therefore, the IBE (identity-based encryption) algorithm was required only in the authentication phase, and the symmetric encryption algorithm was used in the normal data communication phase to reduce the computational pressure.

**Figure 9:**
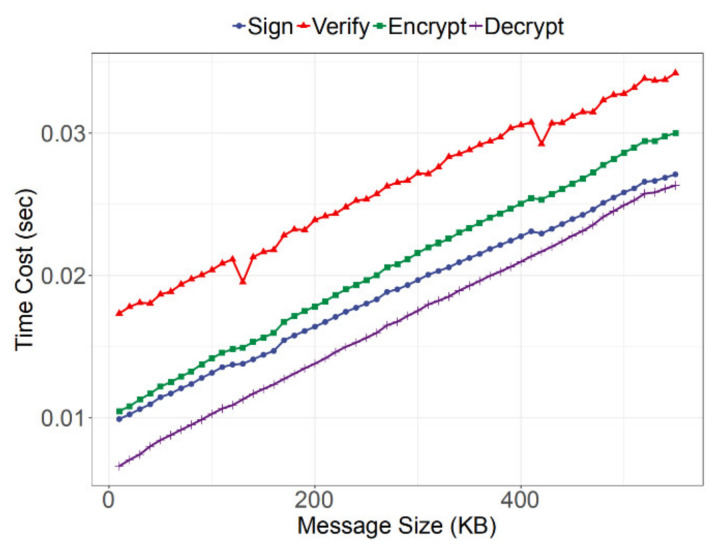
The changes in the running time of each encryption algorithm with different message sizes.

As the size of the message increased, the time of the encrypting algorithm execution increased, too. Figure 9 shows the time cost change trend of different encryption algorithm executions with the different message sizes. It should be noted that because the Linux system dynamically scheduled each thread and adjusted the CPU operating frequency, the time cost of the encrypting algorithm execution did not strictly increase with the increase in data size.

**Figure 10:**
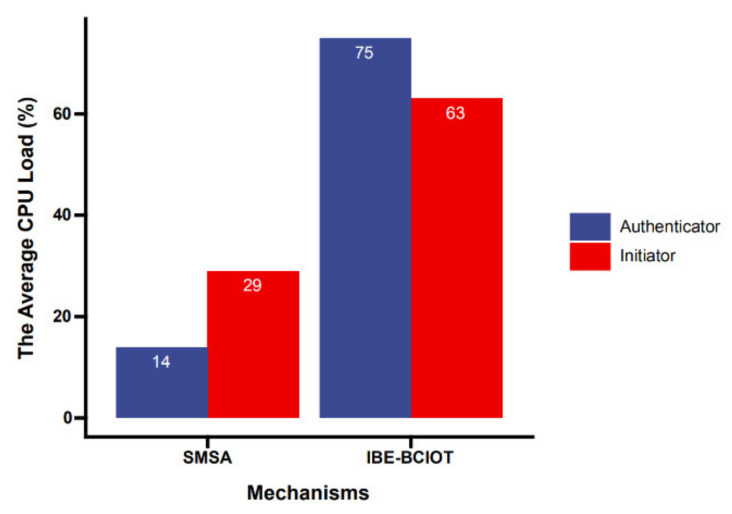
The CPU load of both parties during the identity authentication process.

To further reflect the advantages of the SMSA in terms of computational overhead, we compared the SMSA with the mechanisms IBE-BCIOT [35] under the same experimental settings. Since there are weak terminals in the I-IoT, we mainly compared the computing overhead of the terminal equipment during an authentication process. Initiator and authenticator were both IoT devices. Figure 10 shows the terminal CPU load during a single authentication process. Whether it was the initiator or the identity verifier, the computing performance requirements of the SMSA for the terminal equipment were lower than those of IBE-BCIOT. The test programs were all single-threaded, and the data were single-core CPU load.

Because we allocated most of the encryption calculations to the edge server for execution, the terminal and the server only needed to exchange symmetric encryption keys during the initial authentication process. Table 5 shows the encryption operations that the initiator and authenticator needed to perform during the test. Where SM1 was a symmetric encryption process, the operation speed of its encryption algorithm was much higher than that of the IBE encryption algorithm.

#### 6.2.2. The Performance of Data Exchange

Before the experiment started, we tested the delay between each device in the experimental network with Packet Internet Groper. The test result is shown in Table 6. From the results, since the device was in the local area network, the inherent network delay between each device was at a relatively low level.

We used Python to write a private chain code and deployed it on the BAS in various regions. The private chain used a PoW consensus mechanism. The rule of PoW was to find a number P, and the hash value of the combination of this number and the previous block’s proof would start with four zeros. At the same time, we built a private chain interaction API based on the Flask framework.

At the beginning of the data agent service session, the DAG will authenticate the device, and it will randomly request the device to prove its identity while the session remains connected. Because the times of these authentication operations are limited, it will not be influenced by the normal data exchange between the DAG and entity.

In this case, we mainly focused not only on the time cost of the authentication process. In cross-regional authentication, data reading and writing through blockchain are necessary. Thus, the performance of data reading and writing of the BAS is very important. We recorded the data write delay and data query delay of the BAS in the test. To evaluate the performance of data writing and reading under the different number of BAS nodes, we expanded the number of regions to 10.

**Figure 11:**
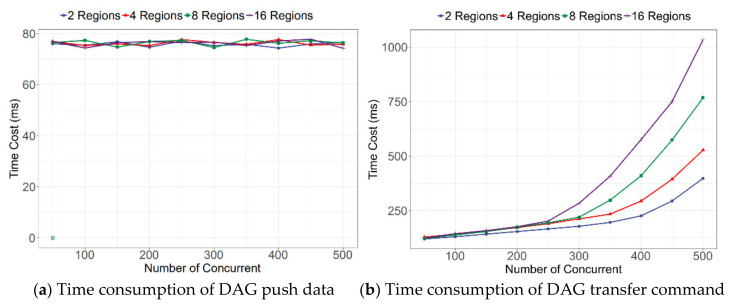
Time consumption of the SMSA data exchange.

From the query delay shown in Figure 11a, the number of BAS nodes basically did not affect the data query time. This is because every BAS in the system will maintain a copy of blockchain data locally. In this case, the data query was executed in the local database without network data exchange.

When the BAS writes data, the delay will increase from low to high as the number of concurrent increases. The more BASs involved, the faster the rise. This phenomenon occurs because the greater the number of BASs, the longer it takes for them to reach a consensus. This problem can be alleviated by optimizing the consensus mechanism. On the other hand, from the combined results in Figure 11a,b, and Table 5, the network delay had a small effect on the performance of entities reading and writing data through the BAS.

## 7. Conclusions

In this paper, to free the sensors and actuators from the various subsystems, we built the sharing mechanism of sensors and actuators for the I-IoT based on the IBC encryption algorithm and combined it with the blockchain. The SMSA deployed the DAG in each region to realize distributed data agency service and through the IMS, R-KGC, and BAS to achieve blockchain-assisted identity-controllable cross-regional secure access. Our contribution can be summarized as follows:

(1) System structure: By deploying the DAG, IMS, and BAS servers in each work region, a safe and reliable distributed sensor and actuator data sharing mechanism was constructed.
(2) Cross-region authentication: The blockchain assisted the IBE to achieve cross-region authentication and maintained the relative independence of the regions.
(3) Standardized interface: by establishing a model of device capability (MDC) we realized standardized access to terminal equipment. This makes it convenient for other users or devices to call the data of the terminal.
(4) Performance evaluation: By setting up a simulation experiment environment, the key technical indicators (communication overhead, computing overhead, etc.) of the proposed mechanism were tested and verified. The experimental evaluation results showed that the communication, computing, and other aspects of the mechanism were within the tolerance of the Industrial IoT.

The SMSA can break the barriers between devices and effectively unearth the potential performance of the Industrial IoT with acceptable computational overhead. In the next phase of research, we will further enhance the security protection capabilities of the SMSA and assist identity authentication by analyzing the data interaction behavior pattern of the entity.

## Figures and Tables

**Table 1:** Classification of mine sensors.

Num	Sensor	Sub-system	Interface
1	Wind speed sensor	Ventilation	RS-485/232
2	Wind pressure sensor	Ventilation	RS-485/232, Analog signal
3	Temperature sensor	Ventilation	Analog signal
4	Carbon monoxide sensor	Ventilation	RS-485/232, Analog signal
5	Bolt stress sensor	Geological surveying	RS-485/232
6	Borehole stress sensor	Geological surveying	RS-485/232
7	RFID personnel positioning	Geological surveying	RS-485/232, Ethernet, WiFi
8	Fire sensor	Monitoring	RS-485/232
9	Infrared camera	Monitoring	Ethernet, WiFi
10	Level sensor	Electromechanical	RS-485/232, Analog signal
11	…	…	…

**Table 2:** The parameters of the IMS, R-KGC, BAS, and DAG.

Equipment	Virtual/Physical	Parameters	Value
IMS	Virtual	CPU Cores	4/AMD Zen3
		CPU Threads	8
		RAM	4 GB
		OS	Ubuntu 20.04
R-KGC	Virtual	CPU Cores	2/AMD Zen3
		CPU Threads	4
		RAM	8 GB
		OS	Ubuntu 20.04
BAS	Virtual	CPU Cores	2/AMD Zen3
		CPU Threads	4
		RAM	8 GB
		OS	Ubuntu 20.04
DAG	Physical	CPU	Intel i7-8550U
		CPU Cores	4/Intel Kaby Lake
		CPU Threads	8
		RAM	8 GB
		OS	Ubuntu 20.04
IoT Device	Physical	CPU	Intel N3700
		CPU Cores	4/Intel Braswell
		CPU Threads	4
		RAM	2 GB
		OS	Ubuntu 18.04.5

**Table 3:** Operational process of encryption algorithms.

Algorithm	Operation Process
Sign	1pair + 1hash
Verify	3pair + 1hash + 1exp
Encrypt	4hash + 2xor + 1pair
Decrypt	2xor + 3hash + 1pair

**Table 4:** Execution time of SMSA on Each entity (unit: s).

Entity	Keygen	Sign	Verify	Encrypt	Decrypt
IMS	-	0.009915	0.017333	0.010468	0.006609
R-KGC	0.000359	0.010152	0.017805	0.010738	0.006800
DAG	-	0.021091	0.032703	0.016139	0.011292
IoT Device	-	0.050324	0.087890	0.053190	0.033821

**Table 5:** Comparison of encryption operations in the identity authentication process.

Mechanisms	Initiator	Authenticator
SMSA	2pair + 2hash + 2SM1	2SM1
IBE-BCIOT	4pair + 9hash + 4xor	5pair + 5hash + 1exp + 2xor

**Table 6:** Communication delay of each device between Region A and Region B (unit: ms).

Entity	$IMS^{B}$	$BAS^{B}$	$DAG^{B}$	$R-KGC^{B}$	$IMS^{B}$	$IoT^{B}$
$IMS^{A}$	3.10	3.29	3.04	3.25	3.09	4.28
$BAS^{A}$	2.95	4.22	3.37	4.06	3.13	5.29
$DAG^{A}$	2.58	1.91	2.15	2.24	1.86	4.34
$R-KGC^{A}$	2.74	3.81	3.66	5.96	4.44	5.06
$IMS^{A}$	3.47	4.98	5.31	3.83	4.57	5.51
$IoT^{A}$	5.05	5.73	5.14	5.60	5.55	6.21

## Data Availability

Correspondence and requests for materials should be addressed to J.L.
